# Color-taste correspondence tested by the Stroop task

**DOI:** 10.3389/fpsyg.2024.1250781

**Published:** 2024-01-24

**Authors:** Yidie Yang, Na Chen, Maiko Kobayashi, Katsumi Watanabe

**Affiliations:** ^1^Faculty of Science and Engineering, Waseda University, Tokyo, Japan; ^2^The Gonda Multidisciplinary Brain Research Center, Bar-Ilan University, Ramat Gan, Israel

**Keywords:** color-taste correspondence, Stroop, congruency effect, sweet-pink, sour-yellow

## Abstract

People consistently associate colors with tastes (e.g., pink-sweet, yellow-sour). However, little has been known on the strength of those color-taste correspondences. The current study examined the congruency effect of color-taste correspondence using two Stroop word categorization tasks. The visual stimuli consisted of food names associated with sweet and sour tastes, presented in different shades of pink and yellow font colors. Participants were instructed to categorize the taste (sweet or sour) of the words in the Stroop word-taste categorization task and to discriminate the font color (pink or yellow) of the words in the Stroop word-color discrimination task. Results showed that participants responded faster in congruent conditions (sweet-pink and sour-yellow) than incongruent conditions (sweet-yellow and sour-pink) in both tasks. Specifically, yellow font colors facilitated the categorization of sour taste words compared to pink font colors, whereas sweet taste words facilitated the discrimination of pink font colors compared to sour taste words. These results provide further evidence for the congruency effect of color-taste correspondence in facilitating the processing of taste-related words and colors. Furthermore, the congruency effect was shown to operate bidirectionally, influencing both the conceptual meaning of tastes and perceptual color perception. This study highlights the significant interference effect of color-taste correspondence on cognitive processing as assessed by the Stroop task.

## 1 Introduction

Taste plays a crucial role in our daily lives, not only for identifying different foods, but also for experiencing the pleasure of gourmet flavors. Taste perception is known to be psychologically sensitive and can be influenced by various factors, such as food color (Spence et al., 2015). Recent studies have found that color can significantly affect how people perceive the taste of foods and beverages, and that different colors can enhance or alter the perception of different taste qualities (Stillman, 1993; Fiszman and Spence, 2015). For example, red and pink colors can enhance sweetness and decrease bitterness, while green and blue colors can have the opposite effect (Maga, 1974; Hidaka and Shimoda, 2014). This process of integrating color perception into the evaluation of taste suggest that humans have developed certain associations between color and taste.

Crossmodal correspondence between color and taste refers to the phenomenon that people consistently associate the basic tastes (i.e., sweet, bitter, salty, sour, and umami) with specific colors (for a review, see: Spence and Levitan, 2021). Previous studies have found that pink and red are commonly associated with a sweet taste; yellow and green are associated with a sour taste; black, green, purple, and brown have been linked to a bitter taste; and blue, white, and gray have been associated with a salty taste (Koch and Koch, 2003; Spence et al., 2015; Woods and Spence, 2016; Saluja and Stevenson, 2018; Chen et al., 2021). These color-taste associations have been observed using both actual food samples and taste words. Saluja and Stevenson (2018) examined color-taste correspondences using actual tastes, and they found that the color-taste mappings were similar to those obtained using taste words. Moreover, color-taste correspondences are observed in different populations and are influenced by cultural and contextual factors (Wan et al., 2014).

Color-taste correspondence can be explained by the statistical learning of co-occurrences in the environment, where people associate certain colors with certain tastes based on the colors of foods and beverages they encounter (Spence et al., 2015; Higgins and Hayes, 2019; Spence, 2019). This learned color-taste mapping information allows for the efficient integration of colors and tastes (Piqueras-Fiszman and Spence, 2011; Spence, 2011; Parise et al., 2013; Chen et al., 2015). For instance, participants made fewer errors in discriminating the taste of real solutions when they were colored according to a color-taste mapping in congruent conditions than incongruent conditions (Zampini et al., 2007). Velasco et al. (2015) examined the congruency effect of color and flavor label of crisp packages using visual search and Go/No-go tasks, and they found that participants responded faster when the color of the package was congruent with the flavor label (e.g., lemon flavor in yellow and tomato flavor in red) than incongruent conditions. However, to our knowledge, no study has examined the congruency effect of color-taste correspondence using the Stroop task.

The Stroop word task is a widely used neuropsychological test that can probe attentional control and cognitive processing to assess the strength and automatic processing of implicit associations, known as the Stroop effect (Stroop, 1935; Richter and Zwaan, 2009; Scarpina and Tagini, 2017). In a classic Stroop task, participants are presented with words that name colors, but whose font color is different. Participants are instructed to either read the word or name the font color, which requires them to inhibit cognitive interference. Cognitive interference occurs when the processing of one stimulus attribute, such as the meaning of the word, influences the concurrent processing of another attribute, such as the font color (Stroop, 1935). The Stroop task relies on the strong overlearned tendency of experienced readers to automatically attend to the meaning of a word, making it challenging to ignore the meaning of the word when instructed to focus on the font color. Longer response times and lower accuracy in naming the font color may indicate difficulties in inhibiting the automatic tendency to categorize the word’s meaning. This interference effect, known as the Stroop cost, is consistently observed and demonstrates the robustness of the task. Thus, the Stroop word categorization task can be a useful tool to test the automaticity and strength of color-taste correspondence, and it has been used in several previous studies investigating the implicit associations (Xiao et al., 2014; Chen et al., 2023).

The current study aimed to examine the congruency effect of color-taste correspondence using the Stroop task. In a previous study, sweet-pink (86.59%) and sour-yellow (80.49%) correspondences were the most chosen associations in a sample of Japanese participants (with a chance level of 20% in Chen et al., 2021). Thus, the sweet-pink and sour-yellow correspondences were selected to be tested using the Stroop task. By manipulating the levels of font color and taste words with congruent and incongruent color-taste pairs, this study examined whether the interference effect of color-taste correspondences would influence behavioral performance with response times and accuracy. Ten words (5 with sweet food names and 5 with sour food names) in ten font colors (5 levels of pink and yellow colors) were presented as visual stimuli. Through the two Stroop word tasks, we explored the strength of sweet-pink and sour-yellow correspondences in both directions with the effect of font color on words taste categorization and the effect of words meaning on font color discrimination. Specifically, participants were presented with color-word stimuli in a modified Stroop task paradigm, where they were required to categorize the taste of the presented words as sweet or sour while ignoring the font colors (Stroop word taste categorization task) and to identify the font color of the presented words as pink or yellow while ignoring the semantic content with taste (Stroop word color discrimination task). We hypothesized that a congruency effect of color-taste correspondence could influence both the taste word categorization and font color processing.

## 2 Methods

### 2.1 Participants

Thirty-eight Japanese undergraduate students (23 males and 15 females, *M*age = 20.4, *SD* = 1.5) took part in this study. All of the participants had normal or corrected-to-normal visual acuity and normal color vision. The sample size was set *a priori* at 33 based on a target of 0.8 power with a medium effect size (Cohen’s *d* = 0.5) using power analysis (G*Power 3.0; Faul et al., 2007). We collected additional data in response to some participants’ poor performance in the task (e.g., error rate > 20%). This experiment was approved by the institutional review board of Waseda University, and conducted in accordance with the ethical standards of the 1964 Declaration of Helsinki.

### 2.2 Apparatus and stimuli

The experiment was programmed in E-Prime 3.0 (Psychology Software Tools^1^). The stimuli were displayed on a 24-inch LCD monitor (EIZO FG2421, EIZO Corp, Hakusan, Japan), with a resolution of 1920 × 1,080 pixels and a refresh rate of 100 Hz. Participants viewed the monitor at a distance of approximately 60 cm.

Ten Japanese words of food name were used as visual stimuli to rank sweetness and sourness. Five of the words were typically associated with two levels of sweetness [high: チョコレート(chocolate), あんこ(red bean paste); low: パフェ(parfait),ドーナツ(donut), 砂糖(sugar)], while the remaining five are associated with two levels of sourness [high: うめぼし(dried plum), グレップフルーツ(grapefruit); low: パイナップル(pineapple), キウイ(kiwi), お酢(vinegar)]. The food names were selected from the top twenty foods based on the ranking of sweet^2^ and sour foods^3^ obtained from the Japanese “Everyone’s Ranking” website. The word stimulus was presented in 40 Pt MS gothic font in the center of the screen. Seven of the words were two to four letters long, and three of the words were in six and eight letters. Sour words are five letters longer than sweet words in total. One Japanese letter sustained about 1.3° of visual angle.

The font colors of the word stimuli were 10 colors rendering gradually from pink (P5 in Table 1) to yellow (Y5 in Table 1). They were grouped as pink font colors (high level pink: P5 and P4; low level pink color: P3, P2, and P1 in Table 1), and yellow font colors (high level yellow: Y5 and Y4; low level yellow color: Y3, Y2, and Y1 in Table 1). The ten colors were measured by PR-655 (Photo Research, Chatsworth, CA, USA), and each color was measured 10 times and the average was calculated. The color information is shown in Table 1.

**Table 1:** Color values in the L^*^a^*^b^*^ color space.

ID	L^*^	a^*^	b^*^	Color
P5	74.99	42.02	−2.64	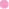
P4	77.44	34.74	4.60	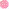
P3	78.59	30.61	10.35	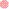
P2	80.10	26.34	14.84	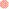
P1	81.27	22.33	20.71	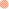
Y1	81.34	20.13	27.77	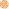
Y2	82.86	15.21	35.58	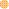
Y3	84.37	11.35	39.72	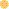
Y4	85.21	9.79	39.88	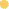
Y5	87.38	2.52	54.28	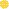

### 2.3 Procedure

**Figure 1:**
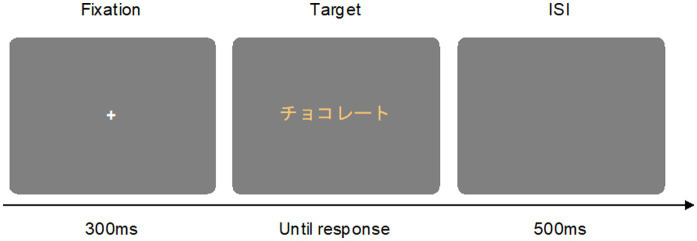
Example of the trial sequence. Participants were asked to categorize the taste of the word stimulus [e.g., “チョコレート(chocolate)”] as sweet or sour by pressing labeled keys in the taste categorization task. Participants were asked to categorize the font color of the word stimulus [e.g., “チョコレート(chocolate)” in yellow font color] as pink or yellow by pressing labeled keys in the color categorization task.

The experiment was carried out in a laboratory with dimmed lighting condition (1 lux on the wall). All participants performed two Stroop word categorization tasks. One was the Stroop word taste categorization task, and participants were asked to categorize the taste of the word stimulus as sweet or sour. The other task was the Stroop word color categorization task, and participants were asked to discriminate the font color of the word stimulus as pink or yellow. At the beginning of each task, written instructions were provided on the computer screen. During the experiment, one of the word stimuli was presented in one of the 10 font colors. The background of the screen was always gray (L^*^ = 20.24, a^*^ = 0.33, b^*^ = 0.36). In the Stroop word taste categorization task, participants were asked to categorize the word stimulus as a “sweet” or “sour” word by pressing a labeled key (e.g., *z* or *m*) on the keyboard with their two index fingers. In the Stroop word color categorization task, participants were asked to discriminate the font color of the word stimulus as pink or yellow by pressing a labeled key (e.g., *z* or *m*). Key assignments were counterbalanced between participants and tasks. Participants were required to respond as quickly and accurately as possible. At the beginning of each trial, a fixation cross appeared for 300 ms, and then a target word stimulus appeared until a response was made. Trials were separated by an inter-stimulus interval (ISI) of 500 ms (see Figure 1). The experimental task had a 2 (taste of words: sweet vs. sour) × 5 (words for each taste group) × 2 (font color groups: pink vs. yellow) × 5 (colors for each font group) × 2 repeated measures design, resulting in a total of 200 trials. Twenty practice trials with feedback preceded the experiment. The main experiment was divided into 5 blocks of 40 trials each. At the end of each block, participants had a self-timed break. Each experiment of the Stroop word categorization task lasted for 5 ~ 10 min. Participants were randomly instructed to either take the Stroop word taste categorization task first or the Stroop word color categorization task first, with a 5-min rest period in between the two tasks. The entire experiment lasted for approximately 20 ~ 30 min.

### 2.4 Data analysis

Data from three participants who made more than 20% errors on either task were excluded. Thus, data from 35 participants were used for the data analysis. Response times (RTs) for correct trials and errors were recorded. Data were analyzed using the R.4.0.2 statistical software (R Core Team, 2020). Statistical analyses on response times and response accuracy were computed with generalized linear mixed-effect model (GLMM) using the function *glmer* from the lme4 package in R (Bates et al., 2015). The distributions of response times are positively skewed and not normally distributed; therefore, generalized mixed-effects models were used to obtain unbiased parameters without the need for data transformation (Lo and Andrews, 2015). We used *fitdistrplus* package to seek the best distribution fit to the present RT data (Delignette-Muller and Dutang, 2015), and selected the inverse gaussian distribution (Johnson et al., 1995). Thus, inverse gaussian distribution with identity link function was specified as the distribution of RTs in the GLMM models. For the error analysis, we used a GLMM with binomial distribution and logit link function. The main interest is the effect of congruency condition (congruent vs. incongruent) and the interactions with levels of taste and font colors on RTs and errors, thus, the fixed factors were congruency condition (congruent vs. incongruent), taste (sweet vs. sour), taste level (high vs. low), font color (pink vs. yellow), font color level (high vs. low), and the interactions. We included random intercept by participant (the random effects were not maximum, so to simplify the model we restricted to a random effect of participant on the intercept for all analysis; Barr et al., 2013; Meteyard and Davies, 2020). To test the main and interaction effects, we compared models with and without that fixed effect of interest using likelihood ratio tests. The *Anova* function (using type III Wald chi-square test) from the *car* package (Fox et al., 2013) was used to test the significance of fixed factors. Significant effects were compared using the *emmeans* package (Lenth et al., 2018). We ran a post-hoc analysis using paired sample *t*-test with Bonferroni correction to determine the simple main effects. Further, Bayes Factors (BF10) were used to determine whether there was support in favor of the alternative (H1) or null (H0) hypotheses (Morey and Rouder, 2018). A value of 1 indicates that null and alternative hypotheses are equally likely, larger values indicate that the data support the alternative hypothesis, and smaller values indicate that the data support the null hypothesis.

## 3 Results

### 3.1 Results of the Stroop word taste categorization task

#### 3.1.1 Response times

**Figure 2:**
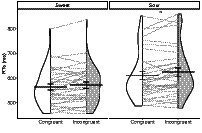
Violin plot showing the distribution of individual RTs in categorizing sweet and sour tasted words in congruent (white color) and incongruent (gray color) conditions. The horizontal line represents the mean. Error bars represent the standard errors of the mean (^**^p < 0.01, Bonferroni corrected).

Error trials (5.31%) and those longer than 3,000 ms and shorter than 100 ms (0.35%) were removed. Trials in which the response times fell out of the outliner (mean RT ± 2.5 sd) for each participant in each color and taste condition were excluded from the data analysis (3.38%). Results of GLMM analysis are shown in Table 2. The GLMM model analysis revealed a main effect of congruency condition, with strong evidence for faster RT for congruent trials (mean RT = 585.16 ms, *sd* = 160.94) than for incongruent trials [mean RT = 595.09 ms, *sd* = 170.76; *x* ^2^(1) = 61.06, *p* < 0.001]. A significant interaction effect between congruency condition and font color was observed, *x*^2^(1) = 63.00, *p* < 0.001. Multiple comparison analysis showed that when categorizing the sour food names, participants responded faster to yellow (congruent condition; 609.38 ms, *sd* = 96.48) than to pink font color [incongruent condition; 623.70 ms, *sd* = 98.90; *t*(34) = 3.02, Bonferroni corrected *p* = 0.0096, Cohen’s *d* = 0.51, BF10 = 7.98]. For categorization of the sweet food names, there was no significant difference in RTs between pink (congruent condition; 563.26 ms, *sd* = 74.65) and yellow (incongruent condition; 571.13 ms, *sd* = 78.09) font colors [*t*(34) = 1.91, Bonferroni corrected *p* = 0.12, Cohen’s *d* = 0.32, BF10 = 0.93; see Figure 2]. There was no significant interaction between congruency condition and levels of font color, *x*^2^(1) = 0.56, *p* = 0.45, and no significant three-way interaction between congruency condition, font color, and levels of font color, *x*^2^(1) = 1.87, *p* = 0.17.

**Table 2:** Summary of fixe effects for RTs in Task 1.

Predictor	Estimate	SE	*t* value	*p*
(Intercept)	595.23	20.60	28.89	<0.001^***^
Congruency	48.94	6.26	7.81	<0.001^***^
Font color	38.17	6.38	5.98	<0.001^***^
Color level	−1.55	5.55	−0.28	0.78
Congruency × Font color	−75.04	9.45	−7.94	<0.001^***^
Congruency × Color level	6.41	8.52	0.75	0.45
Font color × Color level	1.97	8.67	0.23	0.82
Congruency × Font color × Color level	−17.34	12.67	−1.37	0.17

‘^***^’ < 0.001; ‘^**^’ < 0.01; ‘^*^’ < 0.05; ‘^+^’ < 0.1.

#### 3.1.2 Accuracy

Participants made 5.31% of the errors in all trials. The GLMM analysis showed no significant effect of the congruency condition, *x*^2^(1) = 2.30, *p* = 0.13. Thus, there was no significant difference on the effect of font color on taste categorization by error rate.

### 3.2 Results of the Stroop word color categorization task

#### 3.2.1 Response times

Error trials (11.57%) and trials in which response times fell out of the outline (mean RT ± 2.5 sd) for each participant in each color and taste condition were excluded from data analysis (3.44%). There was no trial with response times slower than 3,000 ms and faster than 100 ms.

**Figure 3:**
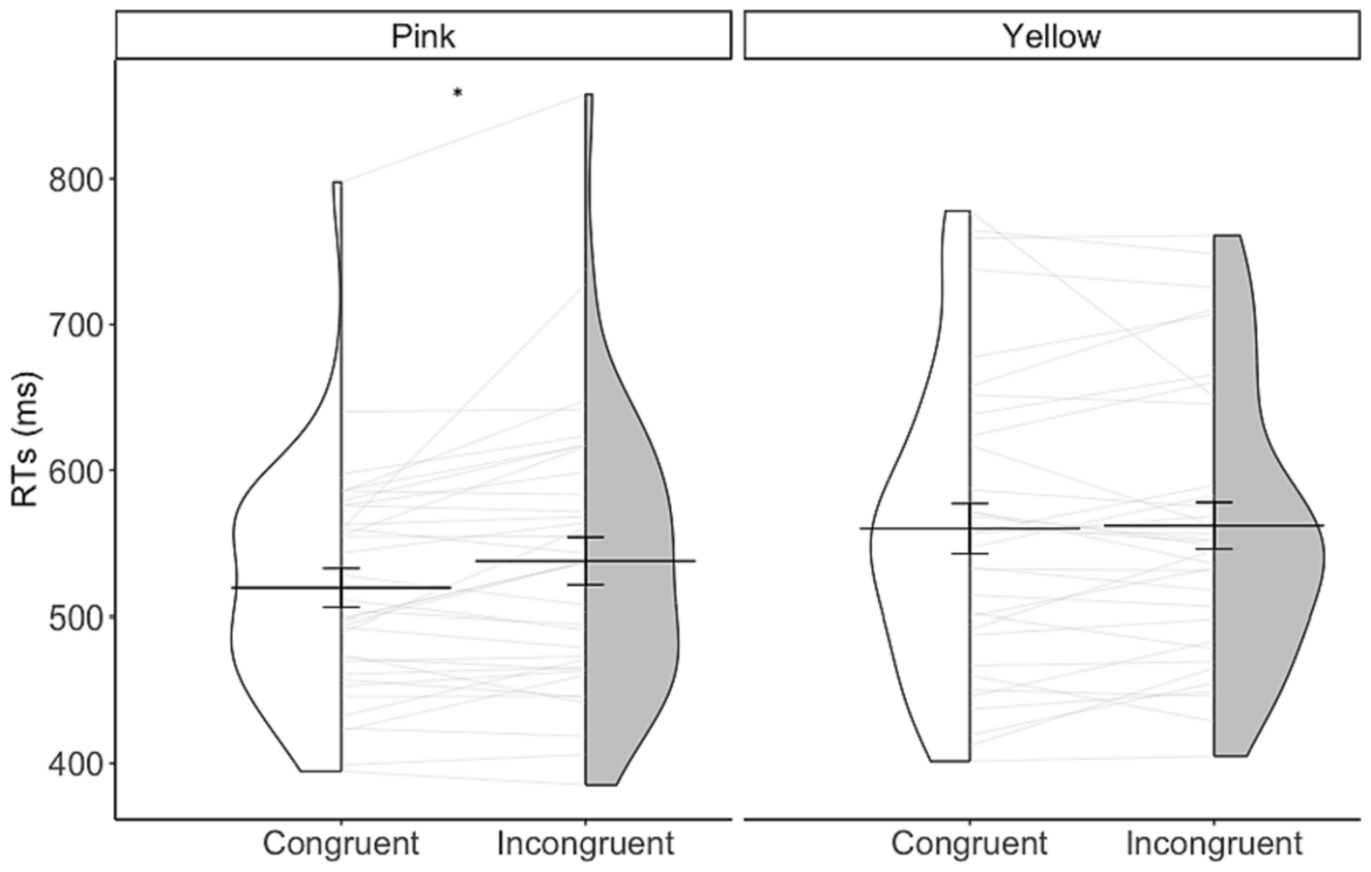
Violin plot showing the distribution of individual RTs in discriminating pink and yellow font colors in congruent (white color) and incongruent (gray color) conditions. The horizontal line represents the mean. Error bars represent the standard errors of the mean (^*^p < 0.05, Bonferroni corrected).

Results of GLMM analysis were shown in Table 3. The model analysis showed a significant main effect of congruency condition, that RTs in congruent conditions (mean RT = 535.93 ms, *sd* = 177.49) were faster than incongruent conditions [mean RT = 549.53 ms, *sd* = 188.47, *x*^2^(1) = 51.42, *p* < 0.001]. A significant interaction effect between congruency condition and taste was observed, *x*^2^(1) = 64.26, *p* < 0.001. Further analysis showed that for discriminating pink font color, participants responded faster in sweet taste words (congruent condition; 519.36 ms, *sd* = 79.13) than sour taste words [incongruent condition; 537.67 ms, *sd* = 96.58; *t*(34) = 2.88, Bonferroni corrected *p* = 0.014, Cohen’s *d* = 0.49, BF10 = 5.93]. For discriminating yellow font colors, there was no significant difference on RTs between sweet (incongruent condition; 561.96 ms, *sd* = 94.59) and sour [congruent condition; 559.95 ms, *sd* = 101.89, taste words, *t*(34) = 0.33, Bonferroni corrected *p* > 1, Cohen’s *d* = 0.06, BF10 = 0.19; Figure 3]. There was a marginal significant effect between congruency condition and level of taste, *x*^2^(1) = 3.64, *p* = 0.056, and a significant three-way interaction between congruency condition, level of taste, and taste, *x*^2^(1) = 6.09, *p* = 0.013.

**Table 3:** Summary of fixed factors for RTs in Task 2.

Predictor	Estimate	SE	*t* value	Pr (>|*z*|)
(Intercept)	547.86	18.79	29.16	<0.001^***^
Congruency	43.23	6.03	7.71	<0.001^***^
Taste	44.58	6.27	7.11	<0.001^***^
Taste level	8.77	5.29	1.66	0.098^+^
Congruency × Taste	−68.96	8.60	−8.02	<0.001^***^
Congruency × Taste level	−14.18	7.43	−1.91	0.056^+^
Taste × Taste level	−22.36	7.82	−2.86	0.004^**^
Congruency × Taste × Taste level	25.64	10.39	2.47	0.01^*^

‘^***^’ < 0.001; ‘^**^’ < 0.01; ‘^*^’ < 0.05; ‘^+^’ < 0.1.

#### 3.2.2 Accuracy

Participants made 11.57% of the error trials, indicating some difficulty in discriminating the low-level pink and yellow font colors. The GLMM model analysis showed no significant effect of congruency condition on errors, *x*^2^(1) = 1.84, *p* = 0.18. Thus, there was no significant difference on the effect of taste on color discriminating by error rate.

### 3.3 Comparison between the two tasks

The GLMM model analysis showed that there was no significant interaction effect between the two tasks and the congruency conditions, on both response times, *x*^2^(1) = 0.13, *p* = 0.72, and errors, *x*^2^(1) = 3.51, *p* = 0.06. Thus, the color-taste congruency effect showed little difference in taste categorization and font color discrimination.

## 4 Discussion

In two Stroop-word categorization tasks, we manipulated the font color (pink and yellow) and the taste of food names (sweet and sour) to examine the congruency effect of associations between color and taste (pink-sweet and sour-yellow). The results showed that color-taste congruency influenced the speed of categorization of both taste-related conceptual words and perceptual font colors. Specifically, yellow font color facilitated the categorization of sour taste words compared to pink font color, while sweet taste words facilitated the discrimination of pink font color compared to sour taste. The high and low levels of taste words and font colors failed to show any interaction effect with the congruency effect. These findings provided further evidence for the congruency effect of color-taste correspondences on cognitive processing and behavioral performance (Velasco et al., 2015; Chen et al., 2021).

When participants were instructed to categorize food names as sweet or sour, the font colors were processed simultaneously, which interfered with the taste process. Our participants responded faster when the font color was congruent with the taste category (sweet-pink/sour-yellow) than when it was incongruent (sweet-yellow/sour-pink). Specifically, sour-tasting words in yellow font colors were recognized faster than those in pink font colors, indicating a congruency effect of sour-yellow correspondences on word taste categorization. Meanwhile, when participants were asked to discriminate the font color as pink or yellow, the associated taste (sweet or sour) of the words was also automatically activated, that discriminating font colors in congruent conditions (pink-sweet/yellow-sour) was faster than in incongruent conditions (pink-sour/yellow-sweet). Specifically, a sweet-related word facilitated the discrimination of pink font color compared to sour-related words, suggesting a sweet-pink correspondence. Thus, congruent color-taste correspondences facilitated perceptual color discrimination. Taken together, the results indicate the congruency effect of an automatically activated color-taste correspondence that modulates the processing of both conceptual word taste categorization and perceptual font color discrimination.

Indeed, it is worth noting that foods in the natural environment that represent sweet and sour tastes often have colors that differ from the commonly associated pink and yellow. For example, dried plum, a popular sour-tasting tsukemono (pickled vegetables) in Japan, is usually red in color. In contrast, chocolate, which is sweet and bitter, are predominantly brown in color. Similarly, donuts, which are known for their sweetness, are typically found in brown and yellow/orange colors. These examples illustrate how learned color-taste correspondences can override natural color associations, potentially influencing the perception and behavior.

Previous research suggests that individuals learn the relationships between sensory features/dimensions across different modalities, leading to the establishment of crossmodal correspondences (Spence, 2011). These correspondences aid in the efficient binding and processing of multisensory information, allowing the brain to efficiently integrate and combine sensory inputs to create a coherent perceptual environment (Parise et al., 2013). In particular, color-taste correspondences can be acquired through mere exposure to the co-occurrence of tastes and colors in foods and beverages (e.g., sweet-pink from the image of ripe and sweet red fruits, and sour-yellow from the yellow lemon). Importantly, studies have shown that similar color-taste correspondences exist across different cultural backgrounds, and some are influenced by cultural contexts (Wan et al., 2014). For example, Japanese individuals show high congruency for sweet-pink (86.59%) and sour-taste (80.49%) correspondences (at a chance level of 20%), which may be due to the regional food behaviors.

Further, color-taste correspondence can also be mediated by the emotional responses from those colors and tastes (e.g., hedonics; Velasco et al., 2015). For example, the color pink is often associated with happiness and romanticism, which aligns with the positive emotions evoked by the sweet taste. Similarly, the color yellow is associated with stimulation and excitement, which aligns with the sensory experience of sour tastes. These overlapping emotional experiences between colors and tastes could contribute to the observed correspondences between them. Thus, the emotional correspondence hypothesis offers a potential explanation for color-taste correspondence.

One limitation of the current study is that it focused only on the sweet-pink and sour-yellow correspondences. Future research could extend this by testing the Stroop effect of correspondences between other basic tastes and colors. Thus, the colors implicitly associated with each basic taste and their strength can be revealed using the Stroop task. Another limitation is the selection of food names representing different levels of sweet and sour tastes from online ranking websites. Future studies could adopt a more systematic approach to examine and rank the basic tastes of different foods to ensure a more comprehensive representation of taste profiles. Moreover, the levels of tastes (i.e., high and low levels of sweetness and sourness) were not well controlled. The main interest of this study is to examine the congruency effect, we did not ask participants to rank the sweetness/sourness of the taste. Future studies could further explore the strength of these associations using the Stroop effect, such that the higher level of color-taste correspondence, the stronger the congruency effect. At last, participants reported whether they have abnormal color vision subjectively, future study should use the Ishihara color vision test and a chemosensory disorder questionnaire to assess the perception of color and taste. Besides, there is no control over the luminance of the chromaticity, future studies need to use isoluminant colors. Our participants were all university students, future studies should consider the effect of age difference on the formation and congruency effect of color-taste correspondence.

In conclusion, the present study demonstrated a congruency effect of color-taste correspondences (pink-sweet and yellow-sour), on both word taste categorization and font color discrimination tested by the Stroop tasks. This highlights the congruency effect of color-taste correspondences in influencing the cognitive processing of perceptual and conceptual information. Future research could investigate the statistical nature of these correspondences by examining the learning effects and training participants to establish color associations with novel tastes.

## Data availability statement

The datasets presented in this study can be found in online repositories. The names of the repository/repositories and accession number(s) can be found in the article/supplementary material.

## Ethics statement

The studies involving humans were approved by The institutional review board of Waseda University. The studies were conducted in accordance with the local legislation and institutional requirements. The participants provided their written informed consent to participate in this study.

## Author contributions

NC coded the experiment. YY and MK conducted the experiments. YY and NC analyzed the data, confirmed the statistical analyses, and drafted the manuscript. KW provided the critical revisions on the introduction and discussion. All authors designed the study, read and approved the final manuscript.

## Footnotes

^1^ http://pstnet.com/products/e-prime/

^2^ https://ranking.net/rankings/best-sweet-foods

^3^ https://ranking.net/rankings/best-sour-foods
